# Missed opportunities for hypertension screening: a cross-sectional study, India

**DOI:** 10.2471/BLT.21.287007

**Published:** 2021-10-27

**Authors:** Sanjay K Mohanty, Ashish Kumar Upadhyay, Prashant Shekhar, Fabrice Kämpfen, Owen O’Donnell, Jürgen Maurer

**Affiliations:** a Department of Population and Development, International Institute for Population Sciences, Govandi Station Road, Deonar, Mumbai 400088, India.; b R4D India project, International Institute for Population Sciences, Mumbai, India.; c School of Economics, University College Dublin, Dublin, Ireland.; d Erasmus School of Economics, Erasmus University, Rotterdam, Netherlands.; e Faculty of Business and Economics, University of Lausanne, Lausanne, Switzerland.

## Abstract

**Objective:**

To assess missed opportunities for hypertension screening at health facilities in India and describe systematic differences in these missed opportunities across states and sociodemographic groups.

**Methods:**

We used nationally representative survey data from the 2017–2018 Longitudinal Ageing Study in India to estimate the proportion of adults aged 45 years or older identified with hypertension and who had not been diagnosed with hypertension despite having visited a health facility during the previous 12 months. We estimated age–sex adjusted proportions of missed opportunities to diagnose hypertension, as well as actual and potential proportions of diagnosis, by sociodemographic characteristics and for each state.

**Findings:**

Among those identified as having hypertension, 22.6% (95% confidence interval, CI: 21.3 to 23.8) had not been diagnosed despite having recently visited a health facility. If these opportunities had been realized, the prevalence of diagnosed hypertension would have increased from 54.8% (95% CI: 53.5 to 56.1) to 77.3% (95% CI: 76.2 to 78.5). Missed opportunities for diagnosis were more common among individuals who were poorer (*P* = 0.001), less educated (*P* < 0.001), male (*P* < 0.001), rural (*P* < 0.001), Hindu (*P* = 0.001), living alone (*P* = 0.028) and working (*P* < 0.001). Missed opportunities for diagnosis were more common at private than at public health facilities (*P* < 0.001) and varied widely across states (*P* < 0.001).

**Conclusion:**

Opportunistic screening for hypertension has the potential to significantly increase detection of the condition and reduce sociodemographic and geographic inequalities in its diagnosis. Such screening could be a first step towards more effective and equitable hypertension treatment and control.

## Résumé

**Objectif:**

Évaluer les occasions manquées de dépistage de l’hypertension dans les établissements de santé en Inde et décrire les différences systématiques de ces occasions manquées entre les États indiens et les groupes sociodémographiques.

**Méthodes:**

Nous avons utilisé les données d’enquête représentatives au niveau national de l’étude longitudinale sur le vieillissement 2017-2018 en Inde pour estimer la proportion d’adultes âgés de 45 ans et plus identifiés comme souffrant d’hypertension et qui n’avaient pas été diagnostiqués comme tels malgré une visite dans un établissement de santé au cours des 12 mois précédents. Nous avons estimé les proportions ajustées selon l’âge et le sexe des occasions manquées de diagnostic de l’hypertension, ainsi que les proportions réelles et potentielles de diagnostic, selon les caractéristiques sociodémographiques et pour chaque État.

**Résultats:**

Parmi les personnes identifiées comme souffrant d’hypertension, 22,6% (intervalle de confiance (IC) à 95% : 21,3 à 23,8) n’avaient pas été diagnostiqués malgré une visite récente dans un établissement de santé. Si ces occasions avaient été concrétisées, la prévalence de l’hypertension diagnostiquée serait passée de 54,8% (IC à 95% : 53,5 à 56,1) à 77,3% (IC à 95% : 76,2 à 78,5). Les occasions manquées de diagnostic étaient plus fréquentes chez les personnes plus pauvres (P = 0,001), moins instruites (P < 0,001), de sexe masculin (P < 0,001), habitant en zone rurale (P < 0,001), hindoues (P = 0,001), vivant seules (P = 0,028) et travaillant (P < 0,001). Les occasions manquées de diagnostic étaient plus fréquentes dans les établissements de santé privés que dans les établissements publics (P < 0,001) et variaient considérablement d’un État à l’autre (P < 0,001).

**Conclusion:**

Le dépistage opportuniste de l’hypertension a le potentiel d’augmenter significativement la détection de cette affection et de réduire les inégalités sociodémographiques et géographiques dans son diagnostic. Ce dépistage pourrait être une première étape vers un traitement et une prise en charge plus efficaces et équitables de l’hypertension.

## Resumen

**Objetivo:**

Evaluar las oportunidades perdidas para el cribado de la hipertensión en los centros sanitarios de la India y describir las diferencias sistemáticas en estas oportunidades perdidas entre estados y grupos sociodemográficos.

**Métodos:**

Se utilizaron datos de encuestas representativas a nivel nacional del Estudio Longitudinal del Envejecimiento 2017-2018 en la India para estimar el porcentaje de adultos de 45 años o más que fueron identificados con hipertensión y que no habían sido diagnosticados con hipertensión a pesar de haber visitado un centro sanitario durante los 12 meses anteriores. Se calcularon los porcentajes ajustados por edad y sexo de las oportunidades perdidas para diagnosticar la hipertensión, así como los porcentajes reales y potenciales de diagnóstico, por características sociodemográficas y para cada estado.

**Resultados:**

Entre las personas identificadas como hipertensas, el 22,6 % (intervalo de confianza del 95 %, IC: 21,3 a 23,8) no habían sido diagnosticadas a pesar de haber acudido a un centro sanitario recientemente. Si se hubieran aprovechado estas oportunidades, la prevalencia de hipertensión diagnosticada habría aumentado del 54,8 % (IC del 95 %: 53,5 a 56,1) al 77,3 % (IC del 95 %: 76,2 a 78,5). Las oportunidades perdidas para el diagnóstico fueron más comunes entre las personas más pobres (*P* = 0,001), con menos educación (*P* < 0,001), varones (*P* < 0,001), de zonas rurales (*P* < 0,001), hindúes (*P* = 0,001), que viven solas (*P* = 0,028) y que trabajan (*P* < 0,001). Las oportunidades perdidas para el diagnóstico fueron más comunes en los centros sanitarios privados que en los públicos (*P* < 0,001) y variaron en gran medida entre los estados (*P* < 0,001).

**Conclusión:**

El cribado oportuno de la hipertensión tiene el potencial de aumentar de manera significativa la detección de la enfermedad y de disminuir las desigualdades sociodemográficas y geográficas en su diagnóstico. Este cribado podría ser un primer paso hacia un tratamiento y control de la hipertensión más eficaz y equitativo.

## ملخص

**الغرض:**

تقييم الفرص الضائعة لفحص ارتفاع ضغط الدم في المرافق الصحية في الهند، ووصف الاختلافات المنهجية في هذه الفرص الضائعة عبر الولايات والمجموعات السكانية الاجتماعية.

**الطريقة:**

استخدمنا بيانات مسح تمثيلية على المستوى الوطني من دراسة طولانية للشيخوخة خلال الفترة 2017 و2018 في الهند لتقدير نسبة البالغين الذين تبلغ أعمارهم 45 عامًا أو أكثر، والذين يعانون من ارتفاع ضغط الدم ولم يتم تشخيص إصابتهم بارتفاع ضغط الدم على الرغم من زيارتهم لمرفق صحي خلال الـ 12 شهرًا الماضية. قمنا بتقدير نسب معدلة حسب العمر والجنس للفرص الضائعة لتشخيص ارتفاع ضغط الدم، وكذلك النسب الفعلية والمحتملة للتشخيص، من خلال الخصائص السكانية الاجتماعية ولكل ولاية.

**النتائج:**

من بين أولئك الذين تم تحديد إصابتهم بارتفاع ضغط الدم، فإن نسبة 22.6% (فاصل الثقة 95%: 21.3 إلى 23.8) لم يتم تشخيص إصابتهم على الرغم من زيارتهم مؤخرًا لمرفق صحي. إذا تم إدراك هذه الفرص، كان من الممكن لانتشار ارتفاع ضغط الدم المشخص أن يزيد من 54.8% (فاصل الثقة 95%: 53.5 إلى 56.1) إلى 77.3% (فاصل الثقة 95%: 76.2 إلى 78.5). كانت الفرص الضائعة للتشخيص أكثر شيوعًا بين الأفراد الأكثر فقراً (نسبة الاحتمال أقل من 0.001)، والأقل تعليمًا (نسبة الاحتمال أقل من 0.001)، والذكور (نسبة الاحتمال أقل من 0.001)، والقرويين (نسبة الاحتمال أقل من 0.001)، والهندوس (نسبة الاحتمال أقل من 0.001)، والذين يعيشون بمفردهم (نسبة الاحتمال أقل من 0.028)، والعاملين (نسبة الاحتمال أقل من 0.001). كانت الفرص الضائعة للتشخيص أكثر شيوعًا في مرافق الصحة الخاصة منها في مرافق الصحة العامة (نسبة الاحتمال أقل من 0.001)، وتنوعت على نطاق واسع عبر الولايات (نسبة الاحتمال أقل من 0.001).

**الاستنتاج:**

إن فحص فرص ارتفاع ضغط الدم لديه قدرة ملموسة على زيادة اكتشاف الحالات، وتقليل التفاوتات السكانية الاجتماعية والجغرافية في تشخيصها. يمكن أن يكون هذا الفحص خطوة أولى نحو علاج وضبط لارتفاع ضغط الدم أكثر فعالية وعدالة.

## 摘要

目的 评估印度医疗卫生机构高血压筛查的诊断错失情况，描述印度各邦和社会人口群组之间错失情况的系统差异。

方法 我们使用印度 2017-2018 年老龄化纵向研究的全国代表性调查数据，估计年龄在 45 岁或以上的确诊患有高血压的成年人以及尽管在过去 12 个月内去过医疗卫生机构却未被诊断患有高血压的成年人的比例。通过社会人口特征数据，我们估计了各个邦调整后的基于年龄-性别的高血压错失诊断比例，以及实际和潜在的诊断比例。

结果 在确诊患有高血压的患者中，22.6%（95% 置信区间，IC：21.3 至 23.8）的患者尽管最近去过医疗卫生机构，却未被诊断出来。如果这些错失机会得到弥补，那么诊断出的高血压患病率将从 54.8%（95% CI：53.5 至 56.1)升高至 77.3%（95% CI：76.2 至 78.5）。错失诊断的情况在较贫穷 (P = 0.001)、受教育程度较低 (P < 0.001)、男性 (P < 0.001)、农村 (P < 0.001)、印度教 (P = 0.00)、独居 (P = 0.028）和参加工作 (P < 0.001) 的人中最为常见。错失诊断的情况在私立医院比在公共卫生机构更常见 (P < 0.001)，并且在各邦之间差异很大 (P < 0.001)。

结论 对高血压进行机会性筛查有可能显著增加该病的确诊情况，并减少其诊断中的社会人口上和地理分布上的不平等情况。这种筛查有可能是迈向更有效和公平的高血压治疗和控制的第一步。

## Резюме

**Цель:**

Оценить упущенные возможности для скринингового обследования артериальной гипертензии (АГ) в медицинских учреждениях Индии и описать систематические различия между ними в разных штатах и социально-демографических группах.

**Методы:**

Для оценки доли взрослых в возрасте 45 лет и старше, у которых выявлена АГ и которым не был поставлен диагноз АГ, несмотря на посещение медицинского учреждения в течение предыдущих 12 месяцев, были использованы данные национального проспективного когортного исследования старения в Индии за 2017–2018 гг. Проведена оценка доли упущенных возможностей диагностики АГ с поправкой на возраст и пол, а также фактической и потенциальной доли диагностированных случаев по социально-демографическим характеристикам и для каждого штата.

**Результаты:**

Тем, у кого была выявлена артериальная гипертензия, 22,6% (95%-й доверительный интервал, ДИ: 21,3–23,8) не был поставлен диагноз, несмотря на недавнее посещение медицинского учреждения. При реализации этих возможностей распространенность установленных диагнозов артериальной гипертензии увеличилась бы с 54,8% (95%-й ДИ: 53,5–56,1) до 77,3% (95%-й ДИ: 76,2–78,5). Упущенные возможности для постановки диагноза более распространены среди более бедного (P = 0,001), менее образованного населения (P < 0,001), среди мужчин (P < 0,001), сельских жителей (P < 0,001), индусов (P = 0,001), одиноких (P = 0,028) и работающих жителей страны (P < 0,001). Упущенные возможности для постановки диагноза чаще встречались в частных, чем в государственных медицинских учреждениях (P < 0,001) и сильно различались по штатам (P < 0,001).

**Вывод:**

Оппортунистическое скрининговое обследование артериальной гипертензии может значительно улучшить выявление этого заболевания и уменьшить социально-демографическое и географическое неравенство при постановке диагноза. Такое скрининговое обследование могло бы стать первым шагом к более эффективному и справедливому лечению и контролю АГ.

## Introduction

Hypertension that is undiagnosed, and so goes untreated and uncontrolled, raises the risks of cardiovascular diseases and premature death.^1^^–^^3^ Failure to prevent ill-health and medical treatments arising from undiagnosed hypertension can strain both health systems and the financial well-being of households. Awareness of hypertension is much lower in low- and middle-income countries than in high-income countries.^4^^,^^5^ In low- and middle-income countries, rates of hypertension diagnosis and management are often lower in socially disadvantaged groups and rural populations.^6^^–^^12^

Improved hypertension screening and management are critical to reaching global targets for reductions in the noncommunicable disease burden, and these improvements can be achieved using highly cost-effective interventions.^13^^–^^15^ Expectation of better health and economic returns on investment in hypertension management that includes detection, diagnosis, treatment and care led to its inclusion in the World Health Organization’s *WHO package of essential noncommunicable disease interventions for primary health care*.^16^^–^^18^ Effective, equitable and easily implementable strategies for early detection of hypertension are key inputs towards improved hypertension management. The package and national guidelines in countries that have adopted it recommend routine assessment of blood pressure for all patients aged 40 years and older who present at a health facility.

In India, estimated deaths related to hypertension increased from 8.9% of all deaths in 1990 to 16.7% in 2018.^19^ With an increase in the population aged 60 years or older, from 101 million in 2011 to 228 million by 2036,^20^ the hypertension disease burden is expected to increase even further. Evidence suggests that 20.6% (12 014/58 400) of adults aged 45 years or older were estimated to have undiagnosed hypertension.^21^ Also, 55.0% (39 737/72 250) of adults 45 years or older used outpatient care and 7.1% (5129/72 250) used inpatient care over the course of a year,^22^ suggesting many missed opportunities to diagnose people during regular health-care visits.^23^ Despite routine opportunistic screening being a natural starting point for improved hypertension treatment and control, it has not yet been universally implemented in India. This study aimed to quantify missed opportunities for hypertension diagnosis in people aged 45 years or older and to describe systematic differences in these missed opportunities across states and sociodemographic groups.

## Methods

### Study design

We used the January 2021 public release of the Longitudinal Ageing Study in India,^22^^,^^24^ which provides nationally representative data on measured blood pressure, reported hypertension diagnosis and treatment and health-care use of older adults in India. From April 2017 to December 2018, the study sampled adults aged 45 years or older and their spouses using a stratified cluster sampling design that covered all states and union territories (states, henceforth), except for Sikkim (further details in the data repository).^25^ A minimum sample size of 1000 participants per state ensured a margin of error of two percentage points at a 95% confidence level in estimating state-specific prevalence of any health condition with a prevalence of 5%.^22^ Samples were larger in more populous states. The weighted sample was representative at state level of the non-institutionalized population aged 45 years or older.

### Measurements and outcomes

Trained enumerators measured the blood pressure of each participant three times using an automatic digital monitor (HEM 7121, Omron Healthcare, Inc., Kyoto, Japan). We used the average of the last two measurements. We classified participants as having hypertension if (i) they had systolic blood pressure ≥ 140 mm mercury (Hg) or diastolic blood pressure ≥ 90 mm Hg; or (ii) they reported ever being told by a medical professional that they had hypertension or high blood pressure and currently taking medication or being under diet and/or salt restriction to control their blood pressure. We defined participants as diagnosed if they reported having been told they had hypertension. All participants were given a health card that recorded their measured blood pressure and other biomarkers, such as height, weight, waist–hip ratio, vision and lung function. Participants with measured blood pressure ≥ 140/90 mm Hg were given a referral letter and advised to go to a health-care provider and for those with blood pressure ≥ 180/110 mm Hg, the enumerator stopped the interview and referred the person immediately to the nearest health centre for further evaluation of their blood pressure and treatment if required.

We identified a missed opportunity for hypertension diagnosis^23^ when a participant had high blood pressure (≥ 140/90 mm Hg), reported not having been diagnosed and reported having visited certain health facilities in the previous 12 months (details in data repository).^25^ We distinguished between missed opportunities at public and private facilities, since participants could report to have visited more than one type of facility during the previous year.

We examined variation in outcomes by sociodemographic factors including years of schooling, age, sex, marital status, working status, living arrangement, caste, religion, rural or urban residence, health insurance cover and household monthly per capita consumer expenditure quintile (hereafter referred to as expenditure quintiles with further details on the expenditure quintiles presented in data repository).^25^

### Statistical analysis

We performed complete case analyses for participants aged 45 years or older. For most analyses, we used participants with hypertension and estimated the proportions who were diagnosed and had a missed opportunity for diagnosis by state and sociodemographic groups. We adjusted these proportions for age and sex differences using the full sample to estimate the age–sex composition of the reference population (details in data repository).^25^ We estimated the proportion of all people with hypertension who had visited a public health facility and yet remained undiagnosed and the respective proportion who visited a private facility. We also estimated the proportion of those with hypertension who would potentially be diagnosed if opportunities to screen and diagnose had not been missed, by adding up the number of participants with a diagnosis and the number of participants with a missed opportunity.

To examine conditional variation in proportions of diagnosis, missed opportunities and potential diagnosis by state and sociodemographic groups, we estimated a multivariable probit model for each of these outcomes and obtained the marginal effect of each covariate averaged across the sample. To quantify the degree of socioeconomic inequality in missed opportunities by expenditure we used a concentration index, that is, the scaled covariance between the outcome and rank of per capita expenditure.^26^ To examine how rates of diagnosis, missed opportunities for diagnosis, and potential diagnosis differed across states and with sociodemographic characteristics of people, we used multivariable models to estimate differences in the likelihood of each of these outcomes occurring. 

We applied sampling weights in all analyses except for the results in Table 1 and took account of stratification and cluster sampling in estimation of confidence intervals (CIs).

**Table 1:** Characteristics of participants aged 45 years or older, participants with hypertension and hypertension prevalence, India, 2017–2018

Characteristic	All participants, no.	Participants with hypertension, no. (%)^a^	Hypertension prevalence, % (95% CI)
**Overall**	58 324	27 124 (100.0)	43.7 (42.8 to 44.6)
**Expenditure quintile^b^**			
Poorest	10 087	3 962 (17.2)	37.0 (35.2 to 38.7)
Poorer	10 483	4 517 (19.0)	41.2 (39.6 to 42.8)
Middle	11 133	5 088 (19.6)	42.7 (41.1 to 44.4)
Richer	12 693	6 199 (20.6)	45.1 (43.3 to 46.9)
Richest	13 928	7 358 (23.6)	52.5 (50.3 to 54.8)
**Education**			
No schooling	27 480	11 959 (47.7)	38.5 (37.4 to 39.5)
0–4 years	6 770	3 195 (11.5)	45.2 (43.2 to 47.3)
5–9 years	13 352	6 387 (21.3)	47.9 (46.5 to 49.4)
≥ 10 years	10 722	5 583 (19.6)	53.3 (51.8 to 54.9)
**Age, years**			
45–54	21 542	7 912 (27.3)	34.3 (33.1 to 35.6)
55–64	18 055	8 644 (30.8)	44.0 (42.7 to 45.4)
65–74	12 976	7 206 (28.5)	52.1 (50.3 to 53.9)
≥ 75	5 751	3 362 (13.4)	54.4 (52.2 to 56.5)
**Sex**			
Male	27 049	12 211 (44.1)	41.4 (40.2 to 42.6)
Female	31 275	14 913 (55.9)	45.8 (44.9 to 46.8)
**Location**			
Rural	38 317	16 184 (64.3)	39.5 (38.6 to 40.4)
Urban	20 007	10 940 (35.7)	53.9 (52.4 to 55.4)
**Caste**			
Scheduled caste	9 895	4 293 (18.2)	40.7 (39.2 to 42.2)
Scheduled tribe	10 183	4 599 (7.6)	38.4 (35.6 to 41.2)
Other Backward Class	22 057	9 918 (45.1)	43.5 (42.1 to 44.9)
Others	16 189	8 314 (29.2)	48.1 (46.7 to 49.5)
**Religion**			
Hindu	42 814	19 180 (80.4)	42.5 (41.6 to 43.5)
Muslim	6 890	3 533 (12.3)	48.9 (45.2 to 52.6)
Christian	5 864	2 856 (3.0)	45.3 (39.8 to 50.8)
Others	2 756	1 555 (4.3)	53.7 (50.2 to 57.3)
**Marital status**			
Married	43 603	19 132 (69.2)	42.6 (41.6 to 43.6)
Widowed	12 838	7 126 (28.4)	47.5 (45.8 to 49.3)
Others	1 883	866 (2.5)	40.4 (34.7 to 46.1)
**Living arrangement**			
Alone	2 094	1 161 (4.6)	46.0 (42.4 to 49.7)
With spouse	8 939	4298 (16.4)	42.1 (39.7 to 44.5)
With children	33 886	14 480 (51.8)	42.8 (41.5 to 44.0)
Others	13 405	7 185 (27.3)	46.6 (45.1 to 48.2)
**Working status**			
Working	27 057	10 628 (40.1)	39.4 (38.1 to 40.7)
Previously worked	15 315	8 306 (31.7)	47.8 (46.5 to 49.2)
Never worked	15 952	8 190 (28.2)	47.2 (45.4 to 49.0)
**Health insurance**			
No	44 841	21 037 (79.3)	43.3 (42.3 to 44.3)
Yes	13 483	6 087 (20.8)	45.2 (43.8 to 46.6)

CI: confidence interval.

^a^ Includes those identified as having hypertension based on measured blood pressure, self-reported diagnosis and reported treatment to control blood pressure.

^b^ The expenditure is the monthly per capita consumption expenditure. More details in the data repository.^25^

Note: Numbers are unweighted, % are weighted.

## Results

Out of a total of 72 250 participants, 65 562 were 45 years or older and of these 58 324 people had complete data. From this sample, 27 124 individuals were identified as having hypertension. Table 1 shows the characteristics of all included participants and of those with hypertension. We estimated that in India, 43.7% (95% CI: 42.8 to 44.6) of adults aged 45 years or older had hypertension. Unadjusted hypertension prevalence was higher among individuals who were richer, better educated, older, female, urban dwellers, in privileged castes and not working.

**Fig. 1:**
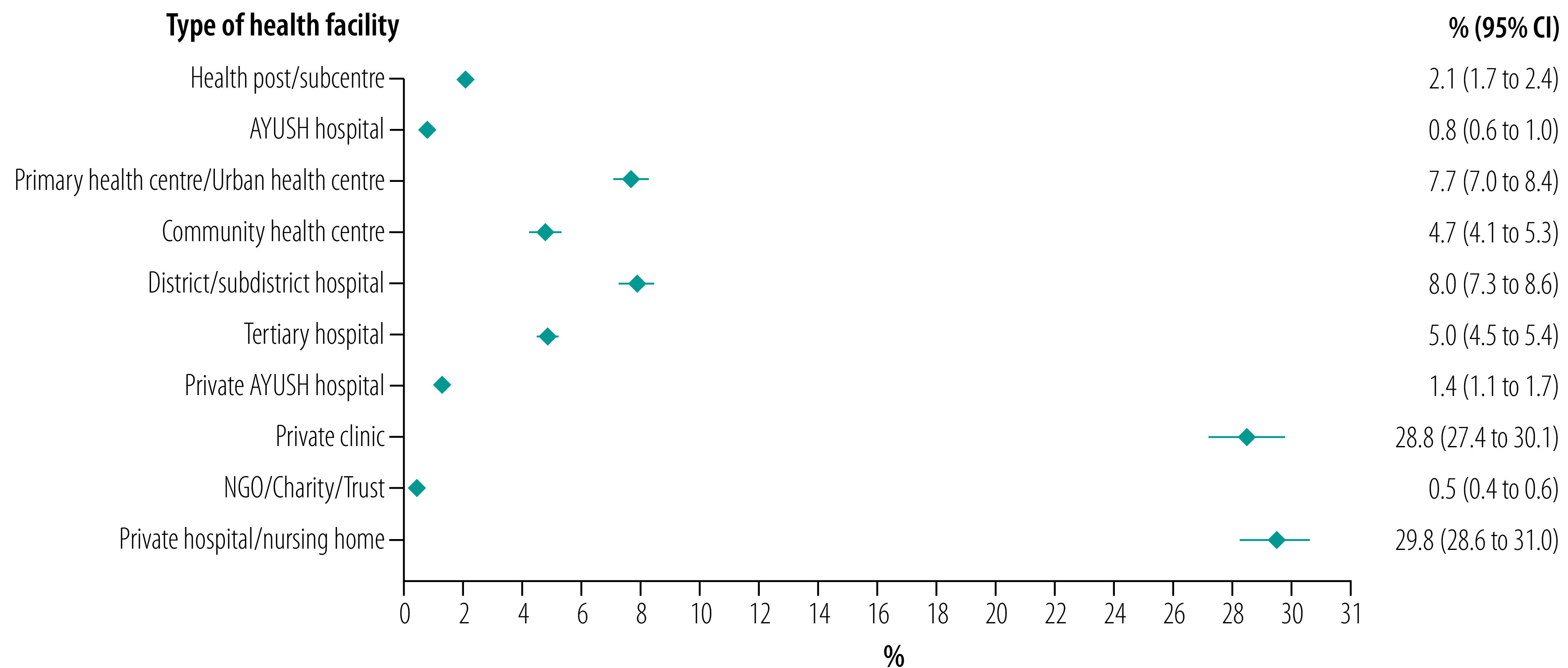
Types of health-care utilization in last 12 months, adults aged 45 years and older with hypertension, India, 2017–2018

AYUSH: Ayurveda, Yoga and Naturopathy, Unani, Siddha and Homeopathy; CI: confidence interval; NGO: nongovernmental organization.

Notes: Percentages of those identified as having hypertension who used each type of health care in the last year. Hypertension identified from measured blood pressure, self-reported diagnosis and reported treatment to control blood pressure.

Among those with hypertension, 64.0% (95% CI: 62.7 to 65.4) had visited a health facility in the last year. Of these, 28.8% (95% CI: 27.4 to 30.1) had visited a private clinic and 29.8% (95% CI: 28.6 to 31.0) had visited a private hospital/nursing home. Utilization of publicly provided health care was substantially lower (Fig. 1).

Table 2 shows age–sex adjusted proportions of people with hypertension who were diagnosed, had a missed opportunity for diagnosis through contact with a health facility, and who potentially could have been diagnosed if opportunities for diagnosis had not been missed. Of people with hypertension, 54.8% (95% CI: 53.5 to 56.1) had been diagnosed. The proportion of people with a diagnosis was significantly (*P* < 0.01) lower for individuals who were poorer, less educated, younger, male, rural dwellers, in scheduled tribes or castes, not married or widowed, and working. Of people with hypertension, 22.6% (95% CI: 21.3 to 23.8) had a missed opportunity for diagnosis at a health facility in the last 12 months. The missed opportunity proportions were higher in the sociodemographic groups with a lower percentage of diagnosed participants. The proportion of those with hypertension who had a missed opportunity for diagnosis at a public health facility was almost half the proportion who had a missed opportunity at a private health facility, 9.0% (95% CI: 8.3 to 9.7) versus 16.7% (95% CI: 15.6 to 17.7). The proportion of missed opportunities at public health facilities was significantly higher for poorer and lower education groups. The socioeconomic gradients in missed opportunities at private facilities were much flatter. The stronger socioeconomic gradient to the disadvantage of the poor at public facilities was also evident from a more negative concentration index of −0.021 (95% CI: −0.029 to −0.014) compared with −0.012 (95% CI: −0.026 to 0.001) at a private facility (further results in data repository).^25^ Of people with hypertension, 5.3% (95% CI: 4.8 to 5.8) had a missed opportunity for diagnosis at a public primary care facility. Individuals who were poorer, less educated, rural and in scheduled tribes or castes were more likely to have used public primary care and had a missed opportunity for diagnosis (data repository).^25^ The proportion of diagnosing people with hypertension could have reached 77.3% (95% CI: 76.2 to 78.5) if opportunities for screening at health facilities had not been missed. As missed opportunities were more common among disadvantaged groups, sociodemographic differences in potential diagnosis proportions were narrower than in actual diagnosis.

**Table 2:** Adults aged 45 years or older with a hypertension diagnosis, missed opportunity for diagnosis or potential diagnosis, India, 2017–2018

Characteristic	Adjusted % (95% CI)				
	Diagnosed	Missed opportunity for diagnosis^a^			Potentially diagnosed^d^
		Total	Public facility^b^	Private facility^c^	
**Overall**	54.8 (53.5 to 56.1)	22.6 (21.3 to 23.8)	9.0 (8.3 to 9.7)	16.7 (15.6 to 17.7)	77.3 (76.2 to 78.5)
**Expenditure quintile,^e^ *P* value**	< 0.001	0.001	< 0.001	0.475	< 0.001
Poorest	43.6 (41.1 to 46.1)	26.0 (23.8 to 28.2)	11.4 (9.9 to 13.0)	18.4 (16.2 to 20.7)	69.4 (67.2 to 71.6)
Poorer	50.1 (47.6 to 52.6)	24.6 (22.5 to 26.7)	11.0 (9.6 to 12.5)	17.1 (15.2 to 19.0)	74.6 (72.6 to 76.6)
Middle	54.6 (52.1 to 57.2)	22.6 (20.8 to 24.5)	9.1 (7.9 to 10.3)	16.6 (14.9 to 18.3)	77.2 (75.0 to 79.4)
Richer	60.2 (58.2 to 62.3)	21.3 (19.6 to 22.9)	8.0 (6.9 to 9.1)	16.2 (14.7 to 17.7)	81.5 (80.0 to 83.0)
Richest	62.2 (60.0 to 64.3)	19.5 (16.6 to 22.4)	6.5 (5.1 to 7.9)	15.4 (12.9 to 18.0)	81.8 (79.7 to 83.9)
**Education, *P* value**	< 0.001	< 0.001	< 0.001	< 0.001	< 0.001
No schooling	47.0 (45.2 to 48.8)	25.5 (23.9 to 27.1)	11.2 (10.1 to 12.3)	18.1 (16.7 to 19.5)	72.0 (70.3 to 73.7)
0 to 4 years	55.6 (52.6 to 58.5)	25.6 (23.2 to 28.1)	10.6 (8.9 to 12.4)	19.3 (17.0 to 21.5)	81.1 (78.9 to 83.4)
5 to 9 years	59.9 (57.6 to 62.2)	21.2 (19.4 to 23.0)	7.8 (6.8 to 8.9)	16.3 (14.7 to 17.9)	80.9 (79.1 to 82.8)
≥ 10 years	67.7 (65.4 to 70.1)	15.5 (13.1 to 18.0)	4.7 (3.7 to 5.7)	12.3 (10.3 to 14.4)	82.9 (81.1 to 84.7)
**Age (years), *P* value**	< 0.001	0.339	0.956	0.049	< 0.001
45 to 54	48.0 (45.5 to 50.6)	23.6 (21.5 to 25.7)	9.2 (8.1 to 10.4)	17.6 (15.9 to 19.4)	71.7 (68.6 to 74.9)
55 to 64	55.0 (52.6 to 57.4)	22.7 (21.0 to 24.5)	8.8 (7.8 to 9.9)	17.1 (15.5 to 18.7)	77.7 (76.0 to 79.4)
65 to 74	59.0 (56.6 to 61.4)	22.1 (20.4 to 23.9)	9.1 (8.0 to 10.2)	16.3 (14.7 to 17.9)	81.1 (79.3 to 82.9)
≥ 75	59.0 (56.1 to 61.9)	21.1 (18.7 to 23.4)	8.9 (6.9 to 11.0)	14.3 (12.2 to 16.3)	80.0 (77.7 to 82.2)
**Sex, *P* value**	< 0.001	< 0.001	0.009	0.003	< 0.001
Male	48.8 (47.2 to 50.5)	24.3 (22.7 to 25.9)	9.9 (8.8 to 11.0)	17.9 (16.5 to 19.2)	73.2 (71.4 to 74.9)
Female	59.5 (57.7 to 61.2)	21.2 (19.8 to 22.6)	8.3 (7.6 to 9.1)	15.7 (14.5 to 16.9)	80.6 (79.2 to 82.0)
**Location, *P* value**	< 0.001	< 0.001	< 0.001	< 0.001	< 0.001
Rural	49.5 (47.9 to 51.1)	25.2 (24.0 to 26.4)	10.3 (9.5 to 11.2)	18.6 (17.4 to 19.7)	74.6 (73.2 to 75.9)
Urban	64.4 (62.6 to 66.2)	17.8 (15.6 to 20.0)	6.6 (5.5 to 7.8)	13.2 (11.4 to 15.0)	82.3 (80.4 to 84.2)
**Caste, *P* value**	< 0.001	0.128	< 0.001	0.003	< 0.001
Scheduled caste	51.9 (49.3 to 54.4)	24.5 (22.6 to 26.5)	12.4 (10.8 to 14.1)	17.2 (15.4 to 19.0)	76.3 (74.3 to 78.4)
Scheduled tribe	36.3 (32.6 to 39.9)	23.2 (20.3 to 26.1)	14.2 (11.7 to 16.6)	12.0 (9.6 to 14.5)	59.5 (56.0 to 62.9)
Other Backward Class	54.9 (53.0 to 56.9)	22.2 (20.2 to 24.3)	8.2 (7.1 to 9.2)	16.6 (14.9 to 18.4)	77.2 (75.4 to 79.0)
Others	61.2 (59.3 to 63.1)	21.7 (20.0 to 23.3)	6.9 (6.0 to 7.8)	17.5 (15.9 to 19.1)	83.0 (81.6 to 84.3)
**Religion, *P* value**	< 0.001	0.001	0.004	< 0.001	< 0.001
Hindu	53.6 (52.1 to 55.1)	23.1 (21.8 to 24.4)	9.4 (8.6 to 10.1)	17.0 (15.8 to 18.1)	76.7 (75.5 to 77.9)
Muslim	60.4 (57.5 to 63.2)	21.4 (17.6 to 25.2)	7.9 (6.2 to 9.7)	16.8 (13.2 to 20.4)	81.8 (79.0 to 84.6)
Christian	54.2 (49.4 to 59.0)	16.8 (14.0 to 19.7)	9.6 (7.0 to 12.1)	8.6 (6.5 to 10.6)	70.8 (66.6 to 75.0)
Others	61.0 (56.5 to 65.4)	20.1 (16.5 to 23.7)	5.8 (3.9 to 7.7)	15.7 (12.5 to 18.9)	81.0 (77.5 to 84.5)
**Marital status, *P* value**	0.009	0.942	0.013	0.078	0.004
Married	55.4 (53.7 to 57.1)	22.7 (21.2 to 24.1)	8.4 (7.6 to 9.2)	17.3 (16.0 to 18.7)	78.1 (76.8 to 79.4)
Widowed	54.0 (51.8 to 56.1)	22.3 (20.4 to 24.2)	10.4 (9.1 to 11.7)	15.1 (13.5 to 16.6)	76.2 (74.0 to 78.4)
Others	45.6 (39.2 to 52.0)	22.7 (17.8 to 27.5)	11.3 (7.7 to 14.8)	15.4 (11.0 to 19.8)	68.2 (62.0 to 74.4)
**Living arrangement, *P* value**	0.054	0.028	0.003	0.181	0.010
Alone	50.4 (45.9 to 54.9)	28.2 (24.0 to 32.5)	14.3 (11.1 to 17.6)	17.1 (13.6 to 20.5)	78.5 (74.9 to 82.1)
With spouse	53.6 (50.8 to 56.4)	22.7 (20.5 to 25.0)	9.1 (7.6 to 10.6)	16.4 (14.6 to 18.2)	76.2 (73.7 to 78.8)
With children	56.1 (54.2 to 57.9)	22.6 (20.8 to 24.3)	8.3 (7.3 to 9.2)	17.5 (15.8 to 19.2)	78.6 (77.4 to 79.8)
Others	53.8 (51.7 to 55.8)	21.5 (19.7 to 23.4)	9.6 (8.4 to 10.8)	15.0 (13.5 to 16.6)	75.1 (72.9 to 77.4)
**Working status, *P* value**	< 0.001	< 0.001	< 0.001	< 0.001	< 0.001
Working	44.5 (42.4 to 46.6)	27.0 (25.0 to 29.0)	10.4 (9.2 to 11.5)	20.2 (18.3 to 22.2)	71.6 (69.8 to 73.4)
Previously worked	59.3 (57.3 to 61.2)	22.5 (21.0 to 24.0)	9.5 (8.4 to 10.7)	16.5 (15.1 to 17.8)	81.9 (80.1 to 83.8)
Never worked	64.3 (62.0 to 66.6)	16.4 (14.6 to 18.2)	6.6 (5.5 to 7.6)	11.9 (10.4 to 13.3)	80.9 (79.2 to 82.5)
**Health insurance, *P* value**	0.232	0.373	< 0.001	< 0.001	0.633
No	54.5 (53.1 to 55.9)	22.7 (21.4 to 24.1)	8.3 (7.6 to 9.1)	17.3 (16.0 to 18.5)	77.2 (75.8 to 78.6)
Yes	56.0 (53.6 to 58.3)	21.9 (20.2 to 23.6)	11.6 (10.2 to 13.1)	14.4 (12.9 to 15.8)	77.8 (76.0 to 79.6)

AYUSH: Ayurveda, Yoga and Naturopathy, Unani, Siddha and Homeopathy; CI: confidence interval.

^a^ We defined missed opportunity as people with hypertension who reported no hypertension diagnosis and used health care in the last 12 months. There were 372 cases that had reported visit to health-care provider but not to a health centre.

^b^ Public health care includes subcentres, primary health centres or urban health centres, community health centres, district or subdistrict hospital, tertiary hospital or AYUSH hospital.

^c^ Private health care includes private hospital or nursing home, private clinic, nongovernmental organization or Church-run hospital or private AYUSH hospital.

^d^ Potentially diagnosed is diagnosed people plus people who have missed the opportunity for diagnosis.

^e^ The expenditure is the monthly per capita consumption expenditure. More details in the data repository.^25^

Note: Sample size is 27 124 participants. Adjusted for age and sex. Unadjusted estimates in data repository.^25^

**Fig. 2:**
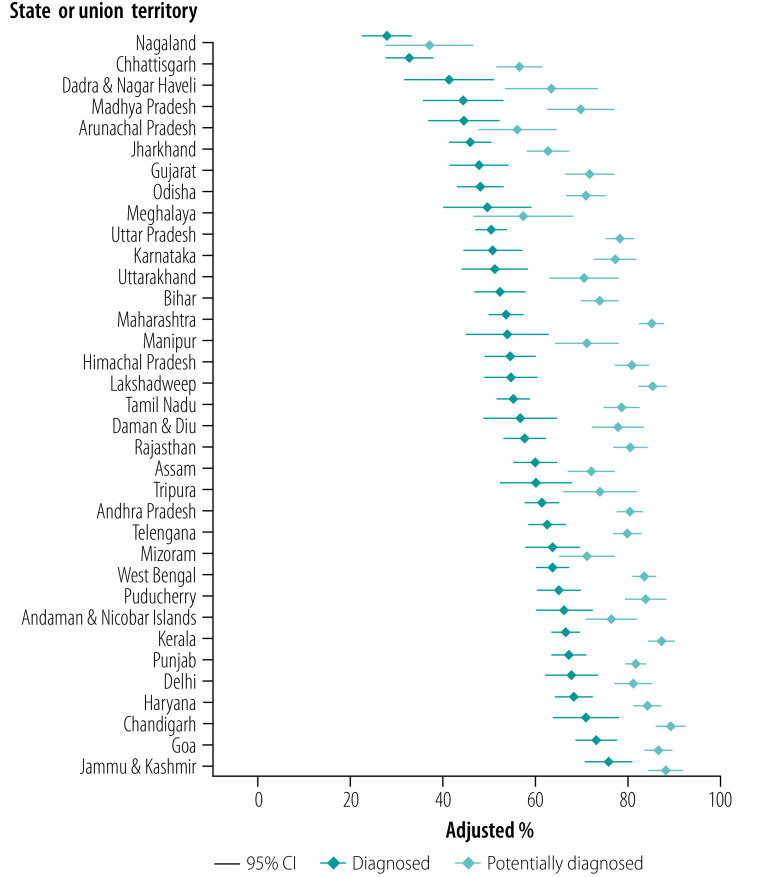
Adjusted percentage of adults aged 45 years and older with hypertension who were diagnosed and potentially diagnosed by state, India, 2017–2018

Fig. 2 shows, by state, the age–sex adjusted proportions of those with hypertension who were diagnosed and the proportions of those who would have been diagnosed if screening opportunities at health facilities had not been missed. States are in ascending order of diagnosed hypertension. 

CI: confidence interval.

Notes: Adjusted for age and sex. Potentially diagnosed is the sum people actually diagnosed and those that could have been diagnosed had they not encountered a missed opportunity. Estimates and unadjusted estimates in table format available in data repository.^25^

Ensuring that people receive hypertension screening at health facilities could substantially raise diagnosis rates in most states. With few exceptions, states with lower proportions of diagnosis generally had higher proportions of missed opportunities. Consequently, between-state inequality in potential diagnosis was lower than between-state inequality in actual diagnosis. The proportion of missed opportunities varied from 7.5% (95% CI: 4.8 to 10.3) in Meghalaya, where health-care utilization was low, to 31.2% (95% CI: 27.8 to 34.5) in Maharashtra, where greater use was made of health care (Table 3). If states eliminated missed opportunities for diagnosis, the ranking of states based on proportion of diagnosis would change. For example, Karnataka, Maharashtra and Lakshadweep would all move from the bottom to the top half of the distribution. 

**Table 3:** Hypertension prevalence and percentage of participants with hypertension who had a missed opportunity for diagnosis, by state, India, 2017–2018

Area	All adults^a^			Adults with hypertension^a^	
	No.	Adjusted hypertension prevalence, % (95% CI)		No.	Missed opportunity for diagnosis, adjusted % (95% CI)^b^
**India**	58 324	43.7 (42.8 to 44.6)		27 124	22.6 (21.3 to 23.8)
**State or union territory**					
Andaman and Nicobar Islands	1 012	64.2 (58.6 to 69.8)		641	10.3 (6.8 to 13.9)
Andhra Pradesh	1 938	56.7 (53.9 to 59.5)		1 068	19.1 (16.5 to 21.8)
Arunachal Pradesh	922	46.5 (41.9 to 51.1)		380	11.1 (4.4 to 17.8)
Assam	1 789	49.2 (45.9 to 52.4)		846	12.1 (9.2 to 15.0)
Bihar	3 181	37.1 (34.6 to 39.5)		1 159	21.5 (18.4 to 24.6)
Chandigarh	761	58.7 (53.5 to 63.8)		439	18.1 (13.1 to 23.0)
Chhattisgarh	1 737	46.0 (42.6 to 49.3)		781	23.5 (20.1 to 26.8)
Dadra and Nagar Haveli	787	45.3 (40.7 to 49.9)		321	21.6 (17.2 to 25.9)
Daman and Diu	753	51.2 (47.5 to 55.0)		375	21.2 (15.2 to 27.1)
Delhi	1 122	52.8 (48.9 to 56.6)		537	13.2 (10.0 to 16.5)
Goa	1 089	58.8 (55.3 to 62.2)		621	13.3 (9.7 to 17.0)
Gujarat	1 807	44.7 (40.8 to 48.6)		776	23.8 (19.5 to 28.1)
Haryana	1 551	47.9 (43.8 to 51.9)		725	15.8 (13.3 to 18.4)
Himachal Pradesh	1 146	52.6 (47.7 to 57.5)		581	26.1 (21.9 to 30.3)
Jammu and Kashmir	1 282	49.5 (45.1 to 53.9)		622	12.1 (7.1 to 17.0)
Jharkhand	2 068	43.1 (40.3 to 45.9)		879	16.7 (13.8 to 19.5)
Karnataka	1 850	44.5 (39.9 to 49.0)		834	26.1 (18.1 to 34.1)
Kerala	1 999	59.5 (56.6 to 62.5)		1 202	20.6 (17.7 to 23.5)
Lakshadweep	943	68.0 (63.1 to 72.9)		640	30.4 (24.3 to 36.6)
Madhya Pradesh	2 431	36.6 (33.5 to 39.7)		865	25.3 (19.7 to 30.9)
Maharashtra	3 026	50.9 (48.1 to 53.8)		1 547	31.2 (27.8 to 34.5)
Manipur	1 087	45.3 (39.7 to 50.8)		503	17.3 (12.5 to 22.1)
Meghalaya	813	51.6 (45.1 to 58.2)		414	7.5 (4.8 to 10.3)
Mizoram	1 004	34.4 (30.2 to 38.7)		361	7.6 (5.1 to 10.1)
Nagaland	1 109	56.7 (46.1 to 67.3)		541	9.0 (3.7 to 14.4)
Odisha	2 367	37.8 (34.9 to 40.8)		902	22.9 (19.6 to 26.3)
Puducherry	1 158	50.5 (47.1 to 53.8)		595	18.7 (15.7 to 21.7)
Punjab	1 758	62.1 (59.6 to 64.7)		1 097	14.3 (11.5 to 17.0)
Rajasthan	1 959	38.2 (35.1 to 41.3)		756	22.7 (18.8 to 26.5)
Tamil Nadu	2 961	45.0 (42.6 to 47.5)		1 435	23.4 (20.3 to 26.6)
Telangana	1 871	51.1 (48.2 to 54.0)		947	17.3 (14.2 to 20.4)
Tripura	934	47.7 (43.9 to 51.5)		428	13.7 (9.7 to 17.8)
Uttar Pradesh	3 881	32.2 (30.0 to 34.5)		1 260	27.8 (24.5 to 31.1)
Uttarakhand	1 176	46.4 (41.8 to 51.0)		555	19.2 (14.6 to 23.8)
West Bengal	3 052	46.5 (43.4 to 49.6)		1 491	19.7 (17.1 to 22.3)

CI: confidence interval.

^a^ Adults 45 years or older.

^b^ We defined missed opportunity as people with hypertension who reported no hypertension diagnosis and used health care in the last 12 months.

Note: Percentages adjusted for age and sex.

The multivariable analysis revealed that, conditional on other sociodemographic controls and state differences, the people in the poorest quintile were 8.0 percentage points (95% CI: 4.7 to 11.3) less likely than the richest quintile to have been diagnosed. The adjusted probabilities of being diagnosed were also lower for individuals who were least educated, younger, male, rural dwellers and in a scheduled tribe. Individuals with health insurance were 3.5 percentage points (95% CI: 0.8 to 6.2) more likely to be diagnosed than uninsured people (Table 4; available at https://www.who.int/publications/journals/bulletin/).

**Table 4:** Likelihood of a difference in hypertension diagnosis, a missed opportunity for diagnosis and a potential diagnosis for adults with hypertension, India, 2017–2018

Characteristic	Percentage point difference (95% CI)^a^		
	Diagnosed	Missed opportunity for diagnosis^b^	Potentially diagnosed^c^
**Expenditure quintile^d^**			
Poorest	−8.0 (−11.3 to −4.7)	0.9 (−2.5 to 4.3)	−7.4 (−10.2 to −4.5)
Poorer	−3.8 (−7.2 to −0.3)	0.1 (−3.4 to 3.6)	−4.1 (−6.9 to −1.3)
Middle	−1.6 (−4.9 to 1.6)	−0.4 (−3.6 to 2.7)	−2.5 (−5.3 to 0.4)
Richer	1.2 (−1.8 to 4.2)	−0.2 (−3.1 to 2.6)	0.7 (−2.1 to 3.6)
Richest	Ref.	Ref.	Ref.
**Education**			
No schooling	−13.6 (−17.0 to −10.2)	8.5 (5.4 to 11.6)	−5.3 (−8.3 to −2.4)
0 to 4 years	−6.1 (−9.9 to −2.3)	8.5 (5.2 to 11.8)	2.6 (−0.5 to 5.8)
5 to 9 years	−5.3 (−8.4 to −2.2)	6.0 (3.4 to 8.7)	0.9 (−2.3 to 4.0)
≥ 10 years	Ref.	Ref.	Ref.
**Age, years**			
45 to 54	−7.8 (−12.1 to −3.4)	2.6 (−0.7 to 5.8)	−5.3 (−9.4 to −1.1)
55 to 64	−1.3 (−4.8 to 2.1)	1.2 (−1.8 to 4.2)	−0.1 (−2.9 to 2.7)
65 to 74	0.5 (−2.6 to 3.6)	1.0 (−1.8 to 3.9)	1.8 (−1.2 to 4.8)
≥ 75	Ref.	Ref.	Ref.
**Sex**			
Male	−11.0 (−13.6 to −8.4)	2.2 (0.0 to 4.4)	−9.2 (−11.6 to −6.8)
Female	Ref.	Ref.	Ref.
**Residence**			
Rural	−8.7 (−11.2 to −6.2)	6.0 (3.6 to 8.4)	−3.1 (−5.7 to −0.6)
Urban	Ref.	Ref.	Ref.
**Caste**			
Scheduled caste	−0.8 (−4.1 to 2.6)	−1.1 (−3.8 to 1.5)	−2.2 (−5.0 to 0.7)
Scheduled tribe	−11.2 (−15.6 to −6.9)	−2.6 (−6.4 to 1.2)	−12.6 (−16.5 to −8.6)
Other Backward Class	0.1 (−2.4 to 2.5)	−2.6 (−4.9 to −0.3)	−2.8 (−5.2 to −0.5)
Others	Ref.	Ref.	Ref.
**Religion**			
Hindu	4.2 (1.2 to 7.1)	−0.6 (−4.4 to 3.2)	3.6 (0.5 to 6.7)
Muslim	3.7 (−0.7 to 8.1)	−4.0 (−7.5 to −0.5)	0.0 (−3.9 to 3.9)
Christian	1.5 (−4.4 to 7.4)	0.2 (−4.8 to 5.3)	1.9 (−3.4 to 7.3)
Others	Ref.	Ref.	Ref.
**Marital status**			
Currently married	11.6 (2.1 to 21.0)	3.6 (−4.5 to 11.7)	17.5 (8.2 to 26.8)
Widowed	7.3 (0.9 to 13.6)	−0.3 (−5.3 to 4.7)	8.2 (1.4 to 14.9)
Others	Ref.	Ref.	Ref.
**Living arrangement**			
Living alone	2.7 (−5.7 to 11.0)	6.9 (−1.4 to 15.2)	9.6 (3.1 to 16.0)
Living with spouse and children	−0.9 (−3.7 to 1.9)	−0.6 (−3.4 to 2.2)	−1.4 (−4.2 to 1.3)
Living with children and others	3.9 (−3.9 to 11.8)	1.9 (−5.2 to 9.1)	6.4 (0.0 to 12.8)
Living with others only	Ref.	Ref.	Ref.
**Working status**			
Currently working	−11.9 (−14.8 to −9.0)	7.2 (4.8 to 9.7)	−5.0 (−7.6 to −2.3)
Ever worked but currently not working	1.4 (−1.9 to 4.6)	4.4 (2.1 to 6.6)	5.3 (2.5 to 8.1)
Never worked	Ref.	Ref.	
**Health insurance**			
No	Ref.	Ref.	Ref.
Yes	3.5 (0.8 to 6.2)	0.1 (−2.0 to 2.2)	3.6 (1.1 to 6.2)

CI: confidence interval; Ref.: reference group.

^a^ We derived percentage point differences from the averaged marginal effects.

^b^ We defined missed opportunity as people with hypertension who reported no hypertension diagnosis and used health care in the last 12 months.

^c^ Potentially diagnosed include both diagnosed people and people who have missed the opportunity for diagnosis.

^d^ The expenditure is the monthly per capita consumption expenditure. More details in the data repository.^25^

Notes: The sample size is 27 124 adults aged 45 years or older. Models also control for state fixed effects. Average marginal effects on missed opportunities at public and private health facilities in data repository.^25^

There were no significant differences in the probability of having a missed opportunity of screening across the expenditure quintiles, although poorer groups had a higher probability of a missed opportunity at a public facility (data repository).^25^ Those with no schooling were 8.5 percentage points (95% CI: 5.4 to 11.6) more likely than those with 10 years or more of schooling to have had a missed opportunity. The likelihood of a missed opportunity was 6.0 percentage points (95% CI: 3.6 to 8.4) higher for those living in rural areas compared with those in urban areas. Other sociodemographic differences in the likelihood of missed opportunities documented in the bivariate analyses were not confirmed by the multivariable analyses. However, these differences were apparent for the probability of a missed opportunity at a public health facility (data repository).^25^ For most sociodemographic characteristics, their associations with the likelihood of potential diagnosis were smaller than their corresponding associations with the likelihood of actual diagnosis (Table 4). For instance, compared to those with 10 years or more schooling, participants with no schooling had a 13.6 percentage point (95% CI: 10.2 to 17.0) lower likelihood of actual diagnosis but only a 5.3 percentage point (95% CI: 2.2 to 8.4) lower likelihood for a potential diagnosis if missed opportunities were eliminated.

**Fig. 3:**
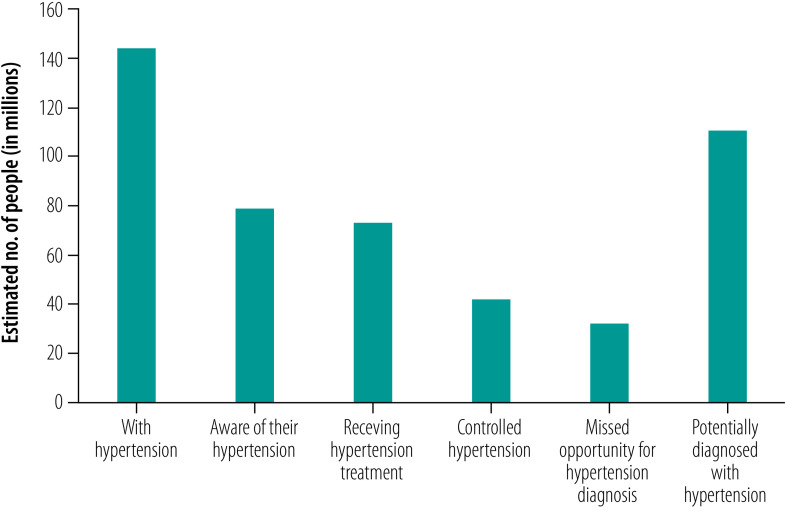
Estimated number of hypertensive cases, diagnosis, treatment and control of hypertension, missed opportunity and potential diagnosis for hypertension in India, 2017–2018

Notes: Potentially diagnosed is diagnosed people plus people who have missed the opportunity for diagnosis

Extrapolating the results of potentially diagnosed people with hypertension to the Indian population aged 45 years or older, we estimated almost a quarter of those with hypertension had missed an opportunity to be diagnosed at a health facility in the previous year. These results translated into around 33 million people with hypertension who could have been diagnosed if routine opportunistic screening at health facilities recommended by national and international guidelines were operating effectively (Fig. 3).^17^^,^^18^^,^^27^ Using the estimates on people treated for hypertension (93%) and having controlled hypertension (53%)^21^ from a published study using the same study population, we predict that 73 million were treated for hypertension, 43 million had controlled their hypertension and 111 million people (of 145 million hypertensive cases) could have been potentially diagnosed if missed opportunities were eliminated (Fig. 3). 

## Discussion

We estimated that 33 million people aged 45 years or older in India had a missed opportunity during a one-year period of having their hypertension diagnosed. Ensuring screening at each health facility visit would raise the proportion of people diagnosed from about 50% to almost 80% in just one year, which is consistent with previous evidence for six Indian states.^23^ The increase in diagnosis rates would also likely result in an increase in the number of people on hypertension treatment and the number of people achieving hypertension control. Such increases would greatly reduce the risk for cardiovascular diseases, which are the largest contributor to the disease burden in India.^28^

Besides documenting the large potential impact of routine opportunistic hypertension screening on overall diagnosis rates, we also showed that this strategy could provide more equitable opportunities for early detection of hypertension.^17^^,^^18^ People who were poorer, less educated, male, rural dwellers, in scheduled tribes or castes, Hindu and working had a higher likelihood of having a missed opportunity for diagnosis. Since these sociodemographic groups also tended to have lower proportions of actual diagnosis, routine opportunistic screening could help close inequalities in diagnosis.^6^^,^^7^ We also observed geographic variation in missed opportunities for hypertension screening with generally higher proportions of missed opportunities in states with lower proportion of diagnosis. Opportunistic screening could, therefore, also narrow between-state inequality in hypertension diagnosis.

Individuals with hypertension who had visited a private health facility in the last year were almost twice as likely to have a missed opportunity for diagnosis compared with those who had visited a public facility. This result reflects the greater utilization of private health care and implies that opportunistic screening would be most effective if it could be implemented in private as well as in public health facilities covered by government guidelines.^27^ Moreover, the high proportion of missed opportunities for diagnosis at public health facilities suggests that implementation of current opportunistic screening guidelines is suboptimal. Substantial improvements in opportunistic screening for hypertension should, in principle, be feasible at all facilities since it requires only standard, low-cost devices. In 2013, the Indian government adopted a national action plan for prevention and control of noncommunicable diseases that aimed to reduce hypertension prevalence by 25% by 2025.^29^ However, the India Hypertension Control Initiative – a programme supported by the government and WHO – which includes opportunistic screening at public primary care and lower secondary care facilities was launched in only five states in 2017.^30^ Our estimates point to the potential impact of such a programme and give urgency to plans to implement it in all states by 2023. The results also suggest that the impact would be even greater if private facilities also implemented screening or if people shifted their health-care utilization towards the public sector.

Our study has limitations. First, like most studies of hypertension awareness, treatment and control based on observational data from a single cross-section, we relied on three blood pressure measurements on a single occasion, rather than multiple occasions, to identify people with hypertension. This approach may have resulted in overestimation of the number of people with hypertension and potential missed opportunities for diagnosis. Second, we could not directly assess whether steps were taken to diagnose hypertension during previous encounters with a health-care provider, because participants were not asked if their blood pressure was measured during their previous visits at health facilities. Third, the lapse of time between visiting a health facility and having blood pressure measured in the survey interview left scope for errors in the classification of missed opportunities. Participants may not have recalled having been diagnosed. Moreover, blood pressure may have been above the hypertension threshold at the time of the interview but not at the time of visiting a health facility. While these potential biases cannot be ruled out, they may be limited given the recency of most of the health-care visits reported – one half of participants reported within a month of the interview and more than three quarters within three months (data repository).^25^ Fourth, our potential diagnosis estimates, based on if all missed opportunities were eliminated, correspond to a hypothetical optimal scenario in which a corresponding opportunistic screening programme would be 100% effective in identifying people with hypertension. In practice, universal blood pressure measurement in all health-care encounters is unrealistic and some cases would be missed. Our estimate should, therefore, be interpreted as a best-case scenario. Finally, our data are three years old and do not capture the most recent circumstances of the Indian health system, notably the disruption caused by the coronavirus disease 2019 outbreak, which is likely to have resulted in even higher proportions of undiagnosed hypertension.

These limitations potentially bias our estimates of missed opportunities for hypertension diagnosis. However, considering that many people with hypertension were likely undiagnosed and that people used health-care facilities to a great extent during our study period, the general finding that opportunistic screening at health facilities would increase the number of people diagnosed most likely holds.

Routine hypertension screening of older adults at public and private health facilities is a promising tool to significantly increase diagnosis rates and reduce socioeconomic and regional inequalities in hypertension awareness and, consequently, its treatment and control in India. Effective implementation of the WHO package of essential noncommunicable disease interventions^16^^–^^18^ and corresponding national guidelines^27^ on opportunistic screening would be an important first step towards reducing the hypertension-related disease burden. To achieve these reductions, all health facilities, especially private facilities, need to adopt the national guidelines on opportunistic screening for adults aged 45 years or older.
